# Performance Prediction of a MongoDB-Based Traceability System in Smart Factory Supply Chains

**DOI:** 10.3390/s16122126

**Published:** 2016-12-14

**Authors:** Yong-Shin Kang, Il-Ha Park, Sekyoung Youm

**Affiliations:** 1 Department of Systems Management Engineering, Sungkyunkwan University, 2066 Seobu-ro, Jangan-gu, Suwon, Gyeonggi-do 16419, Korea; yskang7867@skku.edu; 2 Research Institute of Sustainable Manufacturing System, Korea Institute of Industrial Technology, Cheonan, Chungcheongnam-do 31056, Korea; ihpark0521@kitech.re.kr; 3 Department of Industrial and Systems Engineering, Dongguk University, 3ga, Pil-dong, Jung-gu, Seoul 04620, Korea

**Keywords:** traceability, NoSQL, IoT, smart factory, performance

## Abstract

In the future, with the advent of the smart factory era, manufacturing and logistics processes will become more complex, and the complexity and criticality of traceability will further increase. This research aims at developing a performance assessment method to verify scalability when implementing traceability systems based on key technologies for smart factories, such as Internet of Things (IoT) and BigData. To this end, based on existing research, we analyzed traceability requirements and an event schema for storing traceability data in MongoDB, a document-based Not Only SQL (NoSQL) database. Next, we analyzed the algorithm of the most representative traceability query and defined a query-level performance model, which is composed of response times for the components of the traceability query algorithm. Next, this performance model was solidified as a linear regression model because the response times increase linearly by a benchmark test. Finally, for a case analysis, we applied the performance model to a virtual automobile parts logistics. As a result of the case study, we verified the scalability of a MongoDB-based traceability system and predicted the point when data node servers should be expanded in this case. The traceability system performance assessment method proposed in this research can be used as a decision-making tool for hardware capacity planning during the initial stage of construction of traceability systems and during their operational phase.

## 1. Introduction

In order to maintain competitiveness in the future and respond to intensifying competition in the manufacturing industry, advanced manufacturing countries such as the USA and Germany have formed industry–academic cooperatives such as “Advanced Manufacturing Partnership 2.0” [1] and “Industrie 4.0” [2] in an effort to promote the development and application of smart-factory technology. Smart factories are based on information and communication technologies (ICT) and, more specifically, the Internet of Things (IoT), BigData, artificial intelligence (AI), cyber-physical systems (CPS), and cloud computing [3,4]. In the future, smart factories will evolve into self-adaptive factories where all things will be interconnected, exchanging information, recognizing and assessing situations, and organically fusing the physical world with the cyber world [5,6,7]. It is expected that when future self-adaptive manufacturing environments are realized, manufacturing processes will become flexible, reconfigurable, and adaptable. Therefore, process variations will increase, causing manufacturing and logistics traceability management to become more difficult and complex [8,9].

Traceability is the ability to reconstruct the movement of objects and their environment. It supports process optimization, quality assurance, spill prevention, error proofing, product safety, better pool management, and improved customer service [10,11]. In the past, manufacturing-related traceability sometimes referred to work-in-process (WIP) tracking inside factories. However, the traceability of future connected smart factories will extend beyond the factory and will include the tracking and tracing of raw materials, WIP, and finished products in all stages of the supply chain (receiving, production, processing, warehousing, distribution, selling, etc.). It also includes the tracing of process parameters and temperature/humidity control data collected through smart sensors.

To collect/store/analyze the aforementioned traceability factors in real time, IoT technologies (such as Radio Frequency Identification (RFID), sensors, and GPS) and BigData (including distributed databases and distributed parallel processing frameworks) are essential. Even though BigData research for the manufacturing industry is still in its early stages, applying BigData to production and logistics has become absolutely necessary as RFID and sensor technologies are being increasingly adopted in the manufacturing sector. The reason for this is that manufacturing data collection using IoT sensors produces large volumes of highly complex data. Therefore, BigData is the most appropriate solution to analyze IoT data completely [12].

In recent years, Not Only SQL (NoSQL) databases have been developed and distributed as a replacement for existing relational databases in order to store/process/analyze unstructured high-volume data such as Social Network Services (SNS) and IoT. In handling unstructured high-volume data, NoSQL databases have distinct advantages over relational databases [12,13]. First, NoSQL databases use scale-out architecture that adds low-end resources. This architecture is less expensive than the scale-up methods of relational databases, which replace existing resources with more high-end resources. Second, NoSQL databases ensure consistent scalability even when the amount of data increases owing to the distributed processing of data and computations. Third, because NoSQL data is schema-free and can handle various data sources and formats, it is more flexible than relational databases. For these reasons, NoSQL databases are more appropriate than relational databases for storing a large volume of traceability data collected from different sources. In addition, RFID and sensor data have a low correlation with other data and do not require a high degree of integrity; therefore, they can be stored in NoSQL databases that do not support ACID (atomicity, consistency, isolation, and durability).

Several studies have been conducted on storing IoT data in NoSQL databases. These studies were focused on performance comparison [14], performance improvement [15], and optimal design to an application [13,16,17]. However, no practical research has been reported regarding the achievement of functional requirements and stable operation in real-world business environments using traceability.

Therefore, our research aims to verify the scalability of NoSQL-based traceability systems and proposes a model-based performance evaluation method for stable capacity expansion. To this end, we use MongoDB, a document type NoSQL, as a traceability system database. First, we analyze a traceability data model of MongoDB and a traceability algorithm based on existing research. Secondly, based on the traceability algorithm, we abstractly model the traceability performance for response time at query level. Thirdly, we carry out a benchmark test on the components of the traceability performance model and then specify the model through a regression analysis. Lastly, the traceability performance and scalability are predicted by applying the traceability performance model to an automobile parts supply chain.

## 2. Background and Related Work

### 2.1. NoSQL

Most NoSQL-related research is aimed at performance evaluation and performance comparison with conventional relational database management systems (RDBMSs). In this subsection, studies on NoSQL database performance and NoSQL databases as IoT repositories are introduced.

Nyati et al. [18] tested MongoDB’s performance based on service call numbers and thread numbers when processing massive amounts of data and showed that MongoDB features faster processing times than MySQL from the perspective of read and write. Dede et al. [19] compared the performance of MongoDB’s MapReduce, Hadoop-HDFS’s MapReduce, and Hadoop-MongoDB’s MapReduce (fused with Hadoop as MapReduce and MongoDB as storage). The performance of Hadoop-MongoDB’s MapReduce was shown to be better than that of MongoDB’s MapReduce.

In addition to database performance comparative research, some research was conducted to study the improvement of design and performance. Liu et al. [20] proposed a new data distribution algorithm to solve issues that occur when using MongoDB’s auto-sharding when the data distribution between shards is not even. Kanade et al. [21] compared the performance between embedded design and linking design when normalized data and denormalized data are stored in MongoDB. It was shown through inquiry experiments that 2nd normal form and 3rd normal form data models provide better performance than an un-normal form and the 1st normal form, and that embedded design has better performance than linking design.

Several studies have been conducted on the use of NoSQL to store IoT data. Kang et al. [13] proposed an optimal design of data models and data distribution criteria to store IoT data in MongoDB. Their proposed design utilizes MongoDB to ensure uniform data distribution and better query speed than relational databases. Veen et al. [14] compared the performance of PostgreSQL (an RDBMS), Cassandra (a NoSQL database), and MongoDB as a sensor data repository. It was shown that the usage effectiveness of each product is different depending on data size, data criticality, and read/write performance criticality. Li et al. [15] proposed a massive IoT data management architecture called “IOTMDB.” This architecture constituted not only a method for effectively storing massive IoT data, but also a method for sharing data between different IoT applications based on an ontology. In addition, it defined query syntax for IoT data preprocessing mechanisms, data expressions, and different IoT queries. Le et al. [16] proposed using Cassandra, a column-family type NoSQL database, as a repository for Electronic Product Cord Information Services (EPCIS), which is a GS1 EPCglobal standard for RFID data storing and sharing [22]. Through an experiment, it was shown that, in terms of response time, throughput, and flexibility, the proposed database provides better performance than MySQL-based EPCIS. Li et al. [17] used HBase as GS1 EPCglobal’s Discovery Services (DS) to store external traceability data. This was also shown to have better efficiency and concurrency than relational databases.

### 2.2. Traceability in Manufacturing

There have been several studies on the application of RFID-based traceability in supply chains. Specifically, traceability studies in the manufacturing sector can be classified into data modeling, manufacturing control/algorithms, and RFID-based traceability system development.

In the data modeling area, Jansen-Vullers et al. [23] examined traceability requirements and then defined traceability functions and reference data models that satisfy those requirements. Khabbazi et al. [24] proposed a data modeling method for lot-based traceability systems in make-to-order production environments. They suggested an integrated traceability model including quality lot, quality lot relation, operation lot relation, and order lot by performing conceptual modeling and physical modeling. Ouertani et al. [25] developed a traceability data model and tool to manage distributed product information during the design and manufacturing phases.

Traceability is important for inventory control, WIP management, and scheduling in the manufacturing control/algorithm sector. Chongwatpol and Sharda [26] designed RFID-based real-time scheduling rules applicable to any shop floor. Through simulation tests based on real manufacturing process data, these rules were shown to be better than First In First Out (FIFO) and Earliest Due Date (EDD) methods. Zhong et al. [27] proposed an RFID-based real-time Manufacturing Execution System (MES) framework and applied it to Mass-Customization Production (MCP) shop floors. Moreover, a scheduling algorithm using real-time job pools and rules was developed and shown to allow real-time data WIP control at a certain level. Huang et al. [28] proposed a Petri net-based traceability model and algorithm for manufacturing processes, and developed a prototype information system based on this algorithm. The functionality of this system was confirmed by applying it to the quality control system of China’s honeybee products.

In the system development sector, Huang et al. [29] conducted an initial study on manufacturing traceability using RFID. The authors used RFID technologies in manufacturing reengineering to develop an information system capable of managing the functionality of job shops that fabricate diverse and numerous parts. Sánchez et al. [30] proposed a CPS-based traceability framework for small companies, and designed a traceability system based on cybernetic gloves and cybernetic tables. This traceability system was shown to be better than conventional tag-based traceability systems. Moreover, several studies have been conducted on the development of traceability systems specific to the purpose of each domain [31,32,33].

## 3. Defining Performance Model for a Traceability System

In this section, the data schema and query of MongoDB-based traceability systems are analyzed, and a traceability performance model is defined at query level. Because this research is aimed at evaluating the performance of MongoDB-based traceability systems, we use the data schema and query algorithm of MongoDB-based traceability systems from existing research results.

### 3.1. Traceability Event Schema in MongoDB

**Figure 1:**
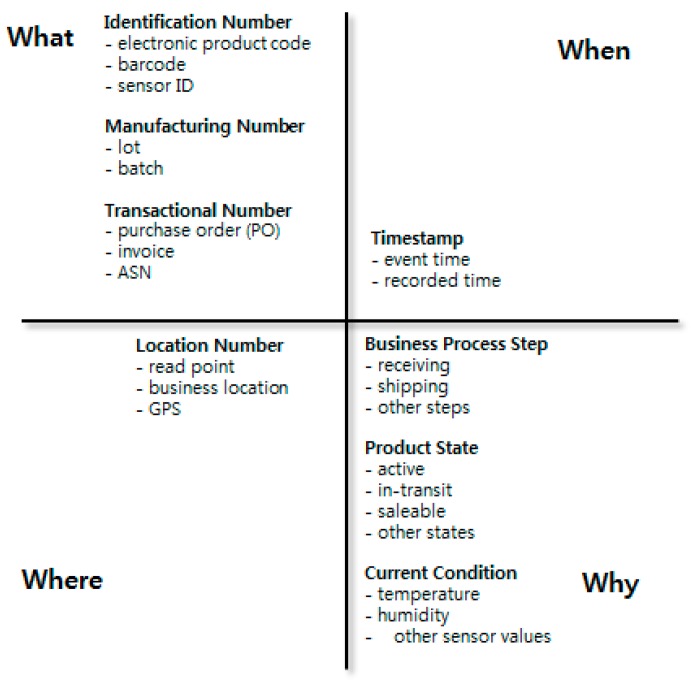
Four dimensions of traceability data.

Traceability data in production and logistics processes must be able to respond to four queries: what, when, where, and why. Figure 1 describes all four dimensions that must be included in a single traceability event based on EPCIS version 1.1 (GS1 EPCglobal) [22]. As proposed in [24], traceability data can include ordering, purchasing, and quality data. However, in this research, the traceability data range is restricted to data that can be collected from IoT, such as RFID, barcodes, sensors, and GPS.

**Figure 2:**
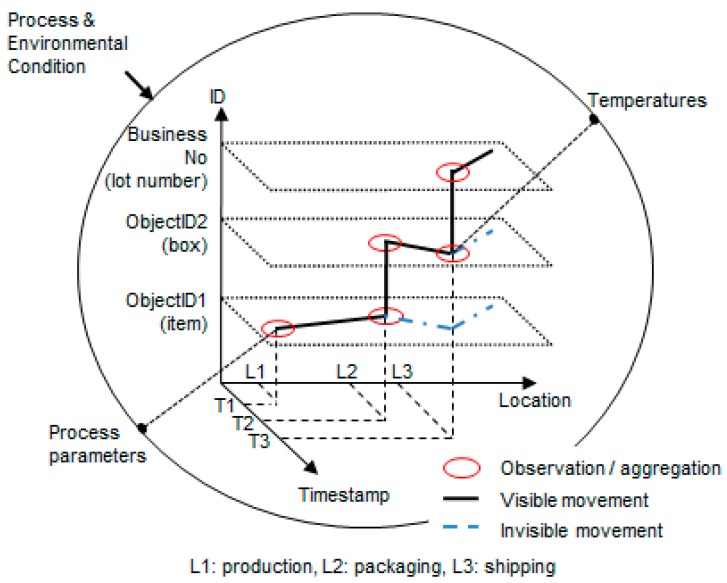
Traceability data (extended from [10]).

In the “What” dimension, each number can be physically or logically combined with other numbers. As shown in Figure 2, ObjectID1 can be packed and moved inside ObjectID2, and they can be connected logically with a business number such as a lot number. In this way, traceability events must include aggregation/disaggregation relationships between components and business numbers of the “What” dimension. In addition, when objects are being processed or are in transit, traceability events can include process parameters and environmental data that can be collected, such as temperature.

**Figure 3:**
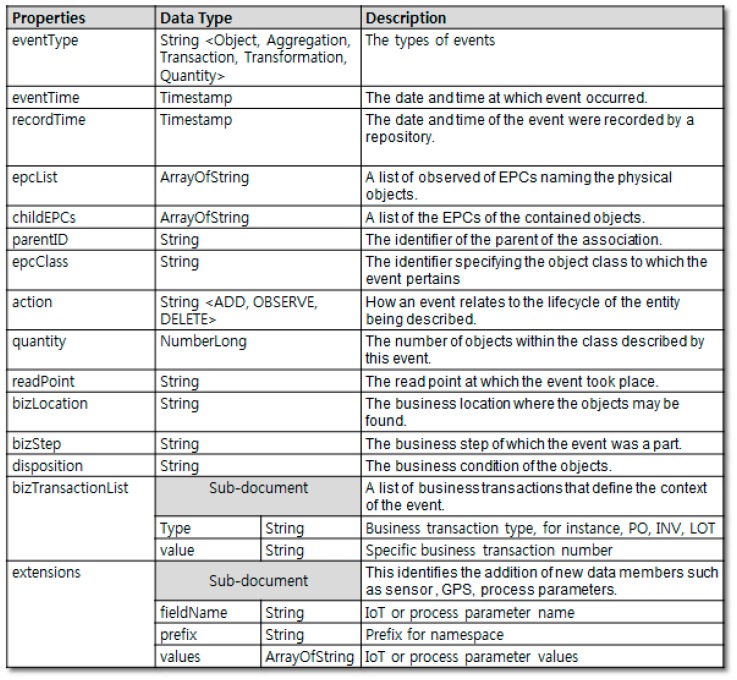
Traceability event data model and example (summarized from [13]).

Considering the work mentioned above in [13], traceability events were modeled as shown in Figure 3. The shard key (data distribution criteria) was selected as (readPoint, eventTime). In this research, the corresponding schema and shard key are used for the MongoDB-based traceability system design.

### 3.2. Traceability Algorithm and Query-Level Performance Model

Although the functional requirements for traceability systems vary based on industry and application, Kang and Lee [10] listed commonly required traceability functions. Among them, we defined a performance model for the pedigree query, the most fundamental traceability function. Then, we considered performance criterion such as response time.

**Figure 4:**
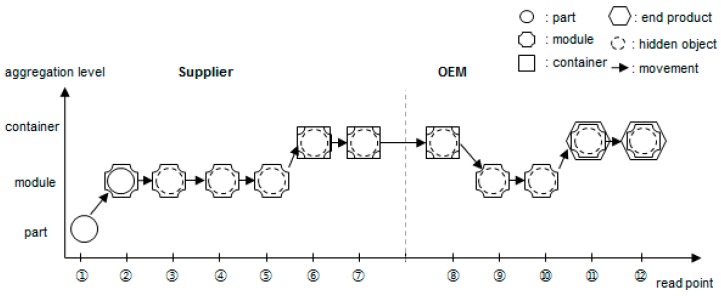
Typical object flow in the supply chain.

The input parameter of the pedigree query is the object identification (ID). All data regarding one object ID cannot be searched simply by inquiring all events of that object ID. As shown in Figure 4, if the part moves invisibly after being assembled in the module, the part movement data is not stored in the database until the disaggregation phase or shipping phase. In other words, if only the tag ID attached to the module is recognized by the reader, the part ID inside the module will no longer be recognized.

**Figure 5:**
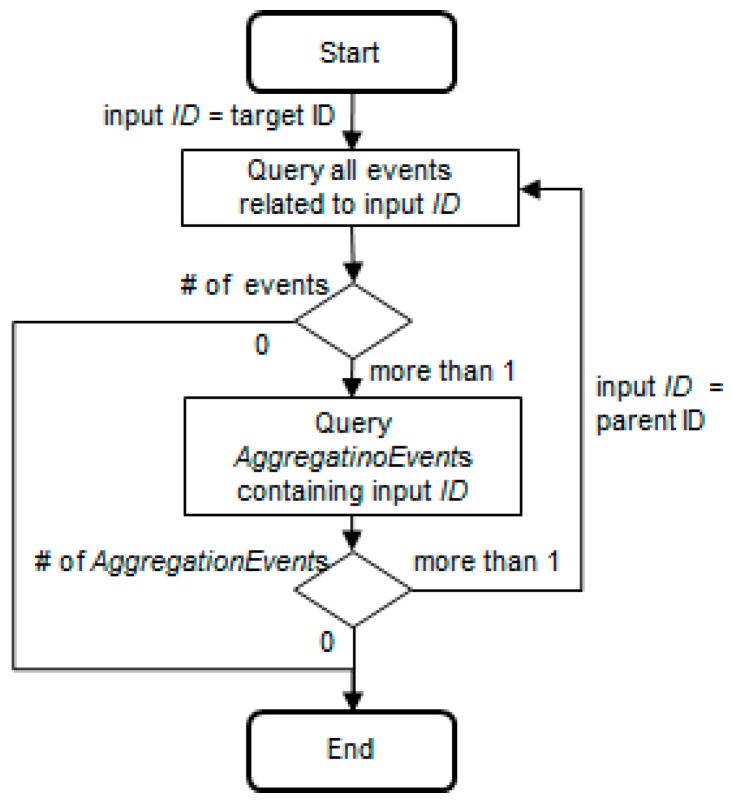
Pedigree algorithm for a site.

Therefore, if the total life cycle of a particular object (part) has to be queried, the object ID (part ID) must be key-tracked first. Then, if that object combines as a child with another parent module, the parent ID must be key-tracked from the aggregation moment until the disaggregation or shipping point of the supplier. In Original Equipment Manufacturer (OEM), if the disaggregation data is all that exists, the parent ID must be key-tracked from the parent module receiving moment until the disaggregation moment. If the parent module combines with other grandparent containers or end products, the grandparent ID must be key-tracked in the same way. Thus, pedigree queries can be classified into two categories: traceQuery (queries all events related to the object ID with input parameters) and aggregationQuery (queries the aggregation relationship of the object ID with another object ID). As shown in Figure 5, if the query algorithm uses recursive methods, it is not affected by the aggregation level.

Thus, $R_{pedigreeQuery}$ (pedigree query response time) is composed of $R_{traceQuery}$ (traceQuery response time) and $R_{aggregationQuery}$ (aggregationQuery response time), and the number of executions of traceQuery and aggregationQuery is determined based on the number of aggregation relationships for one site. $R_{pedigreeQuery}$ is the summation of the response times after executing $R_{traceQuery}$ and $R_{aggregationQuery}$ by all sites that participate in a supply chain. If this is modeled, the following Equation (1) is obtained:

(1) $$R_{pedigreeQuery}=\sum_{n=1}^{k}(a_{n}\times \;R_{traceQuery}+\left(a_{n}+1\right)\;\times \;R_{aggregationQuery}),$$

where n is the participants in supply chain, n = 1…k; and a_n_ is the number of aggregations at each site n.

## 4. Specifying Performance Model with Regression

To specify the query-level performance model defined in the previous section, $R_{traceQuery}$ and $R_{aggregationQuery}$ must be determined. In this section, each query response time is generalized according to the number of data server nodes, data volume stored, and the number of concurrent access clients; all of these factors have the highest impact on service response time.

### 4.1. Benchmark Test

**Figure 6:**
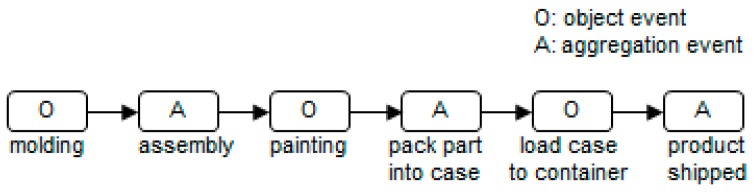
Target process for the benchmark test.

To perform the benchmark test, we prepared a MongoDB cluster composed of six servers with the same specifications. Five of the servers are data-storing servers, and the sixth server was set up as a MongoS and configuration server. The data-querying client program exists in the same network as MongoS, and it was tested in a wired-connection environment. Table 1 indicates the specifications of the servers that form the cluster, and Figure 6 shows the target process used for data generation (the conventional manufacturing process). We assumed that four parts are packed in one case, and cases are loaded into one container.

**Figure 7:**
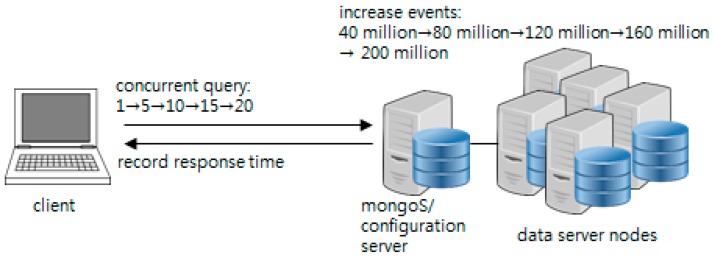
Test scenario.

As mentioned previously, an experiment was performed to measure the response time according to the increasing number of data nodes, number of stored events, and number of concurrent access clients. Figure 7 briefly illustrates the experiment. In this case, the number of data nodes for the initial MongoDB cluster is 1. The steps of the experiment are as follows:

1. Events are generated in accordance with the target process progress and stored in the MongoDB cluster.
2. The client executes traceQuery and aggregationQuery when stored events reach a certain amount (40 million, 80 million, 120 million, 160 million, and 200 million). At this point, the number of concurrent query clients is extended to 1, 5, 10, 15, and 20. Each is queried 30 times, and the response time is recorded.
3. The number of data nodes is increased and repeated from #1 (the maximum number of data nodes is 5).

**Figure 8:**
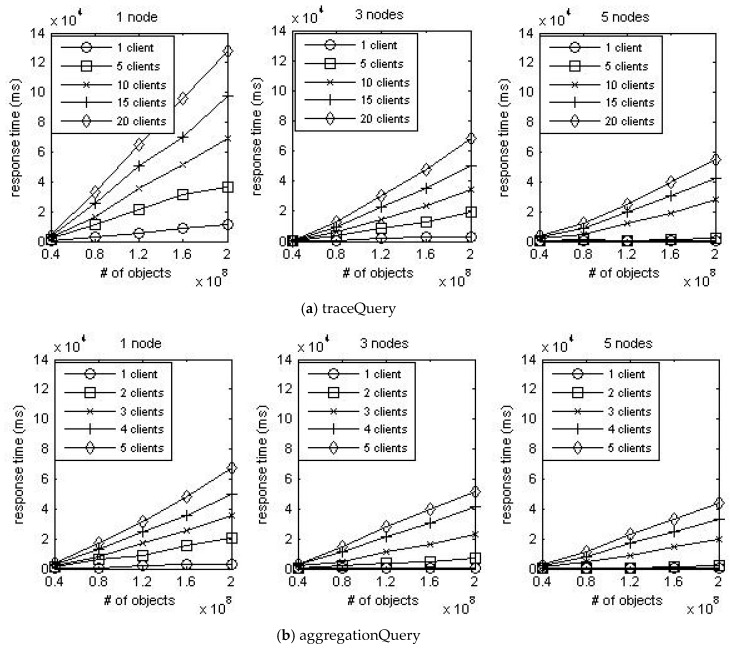
Test results.

Figure 8 shows the result of plotting the mean response time of each query. When the number of data nodes is fixed, the response time increases linearly as the number of stored events increases. In addition, the response time increases constantly as the number of access clients increases. It can be inferred from these results that the increment in the number of data nodes and access clients has a constant impact on increasing response time. Furthermore, the gradient of curves steadily decreases as the number of data nodes increases. It means that the increment in the number of data nodes has a constant impact on reducing response time. Consequently, it can be assumed that all three factors have a linear impact on response time.

### 4.2. Linear Regression

The benchmark test revealed that the number of data nodes, number of stored events, and number of concurrent query clients in the MongoDB cluster have a linear effect on query response times. To derive a linear model, a multiple regression analysis is performed where the number of data nodes, number of stored events, and number of clients are set as independent variables, and response time is set as a dependent variable. The regression equation is as follows:

(2) $$R=\;\beta_{0}+\beta_{1}X_{node}+\beta_{2}X_{volume}+\beta_{3}X_{client},$$

where *X_node_* = number of data nodes; *X_volume_* = number of stored events; and *X_client_* = number of clients.

Table 2 summarizes the coefficients derived through the multiple regression analysis. All *p*-values are lower than 0.05. This shows that the number of data nodes, data volume stored, and number of clients constantly affect the response time of traceQuery and aggregationQuery, and indicates a significant linear relationship.

In conclusion, when the server used in the experiment is installed in a conventional manufacturing process, $R_{traceQuery}$ and $R_{aggregationQuery}$ can be defined as shown in Equations (3) and (4) and can be used as a prediction model:

(3) $$R_{traceQuery}=\;-13634.1-5667.45X_{node}+0.000248X_{volume}+2181.88X_{client},$$

(4) $$R_{aggregationQuery}=\;-15303.2-1934.45X_{node}+0.000178X_{volume}+1310.29X_{client}.$$

## 5. Case Analysis with Simulation

In this section, we describe how the proposed performance model can be applied to a smart factory supply chain through a simulation-based case analysis. We selected automobile parts manufacturing and the logistics business because it is a representative supply chain of the level of multiple aggregations increasing along the supply chain process. We predict the performance change tendency of the pedigree queries according to logistics continuance, and predict the scale-out (server-expand) time and period in which the server can be operated without maintenance procedures, such as server data backup and compression.

### 5.1. Simulation Design

**Figure 9:**
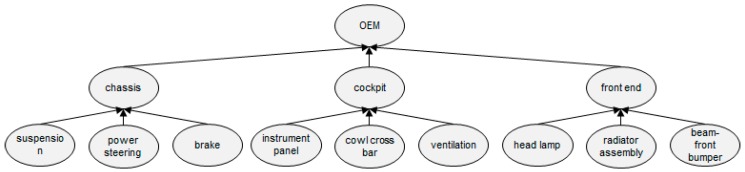
Overall supply chain.

To perform a simulation-based case analysis, the automobile parts production and delivery supply chain designed in [13] (shown in Figure 9) was used. The entire supply chain is composed of a total of 13 workplaces: nine part manufacturers, three module manufacturers, and one OEM.

**Figure 10:**
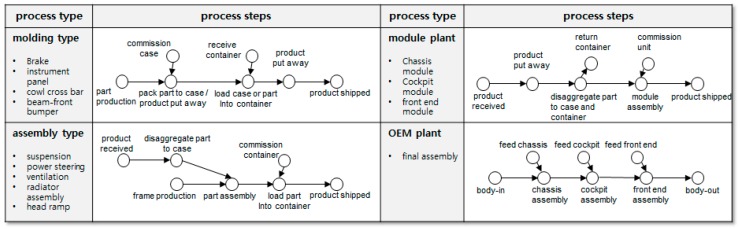
Internal processes according to manufacturing type.

The internal process of each participant is assumed to contribute one manufacturing type (among molding type, assembly type, module plant, and OEM plant), as shown in Figure 10. The molding type, assembly type, module plant, and OEM plant were designed so that two, three, two, and three aggregations occur, respectively.

The simulation parameters (production rate, processing time, and traveling time) of products were equally simplified; details are listed in Table 3. Additionally, temperature data was assumed to be generated every 10 s in all processes, and the transportation stage was set for GPS data to be generated every 10 s. Based on the process model defined here, using these parameters, approximately 100 million events accumulated over two months. When the data was converted into MongoDB, the size of the database grew to approximately 250 GB.

### 5.2. Simulation Results

**Figure 11:**
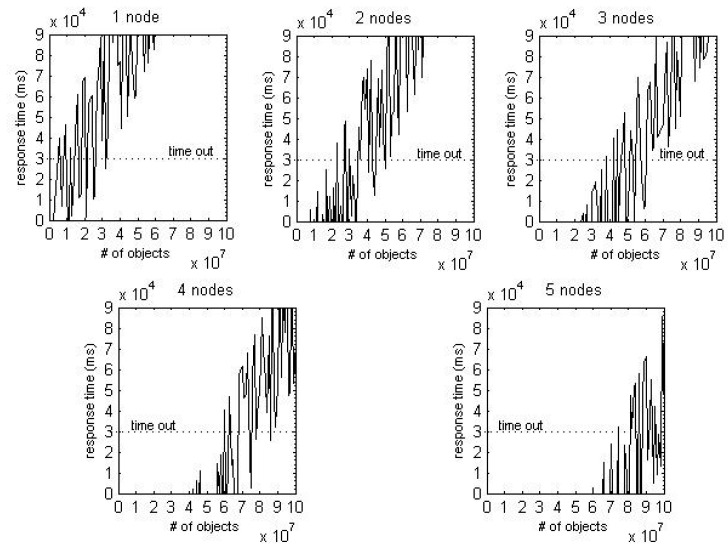
Response time for each cluster.

We assumed that each participant in the supply chain has their own cluster, and their specifications are the same as the cluster that was used for the benchmark test in Section 4. A simulator executed the logistics process (modeled in Section 5.1) and called the performance model of pedigree query (modeled in Section 4.2) for every 1 million events generated. Each query was executed 50 times, and the number of accessing clients was randomly determined according to the uniform distribution U (1,20). Figure 11 shows the average response time according to the number of data nodes regarding pedigree queries based on process continuance.

Assuming that the user can tolerate up to 30 s of response time after executing a pedigree query (i.e., setting the timeout to 30 s), limits on the amount of data for each cluster are generated. These limits are listed in Table 4. For example, clusters composed of 1, 2, and 3 data nodes can store up to 60,000,000, 220,000,000, and 360,000,000 objects, respectively.

Moreover, all subtractions between (n) nodes and (n + 1) nodes are around 150,000,000. The data nodes are linearly required as the number of stored events increases. It means that MongoDB-based traceability system is highly scalable. In conclusion, after first expansion, it is safe to expand the data server node every 150,000,000 increments. Note that if participants use more high-end computing power, response times will be shorter and extension periods will increase. However, it is expected that the tendency of scalability will not be affected by the computing power of a cluster.

## 6. Conclusions

In the near future, smart factories will become a reality. The internal processes of companies participating in supply chains, as well as external processes between companies, will function autonomously and organically as one large system. Traceability data management and stable operation in complex-process environments are becoming key factors that will enable smart factories to achieve a competitive edge in diverse areas such as optimization, product quality, and error proofing.

In this research, a performance prediction model of a traceability system using BigData and IoT, which are key smart factory technologies, was designed to promote the operation of stable and scalable traceability systems. First, traceability requirements and data models of MongoDB-based traceability systems were studied based on existing research. Second, a query algorithm that satisfies a pedigree query (the most common traceability requirement) was analyzed, and a traceability performance model was derived mathematically in terms of response time. Third, a benchmark test was performed considering the number of data nodes, number of stored events, and number of accessing users. Through this test, the traceability performance model was solidified as a linear regression model. Finally, a simulation test was performed to evaluate the traceability performance in automobile parts supply chains. Through the simulation test, we verified that our approach is able to verify the scalability of a MongoDB-based traceability system and to predict a server scale-out point. The difference from previous research [13] reporting a general performance analysis of MongoDB is that a traceability performance is determined by multiple factors that are combined with specific purposed queries such as a location finding and a parent finding.

As a preliminary study, this research contributes to defining methods for stable traceability operations, one of the primary business requirements in the manufacturing sector, where BigData and IoT technologies are starting to be applied. The MongoDB-based traceability system performance prediction method proposed in this research will serve as a decision-making tool for predicting the required number of servers during the initial construction of traceability systems, and the point when servers should be added. In addition, by showing that existing traceability algorithms can evolve in better computing environments, it will become a useful resource for researchers trying to carry out similar studies.

In the future, further research will be required on performance models for pedigree queries, as well as on traceability functions that are commonly used in the manufacturing industry, such as lot tracking and Bill of Material (BoM) exploring. Furthermore, in terms of scalability, this research used only the response time as a performance indicator to build the model. However, it will be important to conduct further research on performance models that consider other perspectives such as availability, reliability, and accessibility, as well as other indicators such as latency, throughput, transaction time, and execution time.

## Figures and Tables

**Table 1:** Specification of servers.

CPU	RAM	HDD	OS
2.53 GHz × 8	16 GB	500 GB	Ubuntu Server 12.04

CPU: Central Processing Unit, RAM: Random Access Memory, HDD: Hard Disk Drive, OS: Operating System.

**Table 2:** Regression results.

	Coefficient	Regression Coefficient					Correlation Coefficient		
		$\beta_{0}$	$\beta_{1}$	$\beta_{2}$	$\beta_{3}$		Standard Error	T Statistics	*p*-Value
traceQuery	0.72	−13634.1	−5667.45	0.000248	2181.88	$X_{1}$	991.65	−5.71516	0.0000002390428
						$X_{2}$	0.000028	8.66	0.000000000001
						$X_{3}$	238.35	9.15	0.0000000000001
aggregationQuery	0.77	−15303.2	−1934.45	0.000178	1310.29	$X_{1}$	526.36	−3.67517	0.000458002579347691
						$X_{2}$	0.000015	11.69	0.000000000000000003
						$X_{3}$	126.51	10.36	0.000000000000000772

**Table 3:** Simulation variables. (unit: min).

Production Rate		Movement Time		Work Time	
part	1/min	part	N(1,0.2)	part packaging	N (1,0.2)
case	(1/3)/min	case/container	N(5,0.2)	case loading	N (1,0.2)
container	(1/15)/min	container waiting	N(1,0.2)	container loading	3/min

N: Normal Distribution.

**Table 4:** Maximum amount of data stored within the timeout.

# of Nodes	1 Node	2 Nodes	3 Nodes	4 Nodes	5 Nodes
# of objects	60,000,000	220,000,000	360,000,000	610,000,000	750,000,000
subtraction		160,000,000	140,000,000	150,000,000	140,000,000
